# The Development of Disinhibited Social Engagement Disorder: A Case Report on the Psychiatric Implications of Prenatal Fentanyl Use

**DOI:** 10.7759/cureus.61078

**Published:** 2024-05-25

**Authors:** Ali Golparvar, Omar Nafeh, Meena Alzamani, Victoria Singh, Marilena Jennings

**Affiliations:** 1 Child and Adolescent Psychiatry, BronxCare Health System, Bronx, USA; 2 Psychiatry, American University of the Caribbean, Bronx, USA

**Keywords:** integrative approach to attachment disorders, integrative approach, integrative approach to adhd, child psychiatry, prenatal fentanyl's influence

## Abstract

Exposure to drugs during pregnancy can result in neonatal abstinence syndrome (NAS), low birth weight, attention-deficit/hyperactivity disorder (ADHD), and behavioral issues, particularly during the school-age years. Recent research has expanded our understanding of the consequences of fentanyl exposure during pregnancy beyond the more commonly recognized effects, including respiratory complications, neurodevelopmental effects, increased risk of substance use disorders, gastrointestinal complications, cardiovascular effects, epigenetic changes, behavioral and emotional regulation disruptions, and long-term cognitive impairments. We present the case of a five-year-old female placed in foster care with a past medical history of asthma and a past psychiatric history of ADHD, oppositional defiant disorder (ODD), and disinhibited social engagement disorder. Her mother has a long history of substance use during pregnancy. From our interaction with the patient presented, we see that prenatal exposure to substances such as fentanyl and the disruption of attachment figures can have profound and lasting effects on a child's life, encompassing behavioral and cognitive aspects.

## Introduction

This report examines the various factors that can have a substantial impact on the health and mental well-being of children during their growth and development. The ongoing opioid crisis requires the need to understand the long-term implications of various factors in a child's physical development and, as we discuss, behavioral development. Specifically, this case study examines the relationship between prenatal fentanyl exposure, attachment disorders, and attention-deficit/hyperactivity disorder (ADHD) in a patient experiencing challenges at the intersection of these influences. While a developing child possesses a remarkable capacity to adapt to the environment and circumstances met during their formative years, the adaptive behavioral responses may sometimes diverge from conventional social standards. This exploration sheds light on the profound and lasting effects that prenatal substance exposure and disrupted attachment figures can have on a child's life, particularly in terms of their behavioral and cognitive development, emphasizing the urgent need for a multidisciplinary approach to mitigate adverse outcomes. The ongoing opioid crisis demonstrates the importance of understanding not only the long-term implications on the user but also the behavioral effect on the unborn child if applicable. The patient highlighted in this report is a five-year-old female with a running diagnosis of ADHD, oppositional defiant disorder (ODD), and disinhibited social engagement disorder.

## Case presentation

This is a case regarding a five-year-old female in kindergarten with a past medical history and psychiatric history of asthma and ADHD, respectively. When the patient was born, fentanyl and a multitude of other drugs were detected in her system. Her mother was considered unsuitable by the Administration for Children's Services (ACS) due to a positive drug test, along with the father, who nearly dropped the child after falling asleep, presumably under the influence of substances. As a result, she was removed from her birth parents' care and placed in foster care. Before entering the foster care system, the patient had to spend multiple weeks in the neonatal intensive care unit (NICU) for the detoxification of fentanyl and other drugs from her system. The social worker involved in this case noted that she was placed with a foster family after being discharged at 18 months. At the age of 18 months old, she was formally adopted by her birth mother's cousin; however, in November of 2022 following a physical abuse case, she was then placed with a new foster mother, who she now refers to as "grandmother," who has been taking care of here until the patient's presentation at the hospital.

The patient presented to the Comprehensive Psychiatric Emergency Program (CPEP) brought by emergency medical services from school due to aggressive and hyper behavior. Her electronic medical records indicated that she had been admitted to CPEP five days prior to this visit for similar symptoms. On the examination, inconsistent eye contact was made, and most questions were not answered. The patient was hyperactive, requiring multiple redirections, and called many nurses and medical students "mommy," demonstrating insecure attachment with disinhibited social engagement disorder. Her teachers at school described her as destructive, grabbing and throwing objects, specifically scissors, and being disruptive continuously throughout the day. When asked, she denied having any suicidal or homicidal ideation and denied having any hallucinations. Once admitted to the hospital, she demonstrated signs of hyperactivity and aggression, as she was seen spitting and hitting nurses and patient care technicians when redirected and was constantly running away from staff.

Before admission, the patient was on 2.5 mg of methylphenidate prescribed by her school's therapist with minimal response. Currently, she is on 5 mg methylphenidate and still displays severe hyperactivity and signs of oppositional defiant disorder when verbally redirected. In the hospital's inpatient school, the teacher reports that she is easily redirected with rewards, while before admission, she displayed continuous aggression and hyperactivity in her school. Her primary psychiatric diagnosis based on her presentation was the following: combined-type, moderate ADHD with other running diagnoses of disinhibited social engagement disorder of childhood versus oppositional defiant disorder.

Her treatment plan included 5 mg methylphenidate twice a day, which can be titrated up until symptomatic improvement. Upon discharge, the methylphenidate was titrated up to 18 mg along with other medications as shown in Table 1. The expected outcome goal is to improve patient safety, decrease impulsivity and hyperactivity, and decrease aggression caused by redirection.

**Table 1:** Treatment Plan PRN, as needed; q, every

Initial Inpatient Treatment Plan	End Inpatient Treatment Plan	Discharge Medications
Methylphenidate 5 mg q 7 am and q 12 am	Clonidine 0.05 mg q 6 am and 0.1 mg q 2 pm and q 8 pm	Clonidine 0.05 mg q 6 am and 0.1 mg q 2 pm and q 8 pm
Chlorpromazine 10 mg PRN for agitation	Methylphenidate HCl immediate-release (Ritalin) 5 mg q 24 hours at 6 am	Methylphenidate HCl immediate-release (Ritalin) 5 mg q 24 hours at 6 am
Diphenhydramine 25 mg q eight hours PRN for agitation	Methylphenidate ER (Concerta) 18 mg q 24 hours at 6 am	Methylphenidate ER (Concerta) 18 mg q 24 hours at 6 am
	Risperidone 0.25 mg q 24 hours at 8 pm	Risperidone 25 mg at 6 am and 8 pm
	Diphenhydramine 25 mg q eight hours PRN for agitation	
	Chlorpromazine 10 mg q eight hours PRN for agitation	

## Discussion

Several needs of a developing child need to be met to ensure a healthy upbringing. The development of a child has the remarkable capacity to conform to the change of its environment and the circumstances it encounters; however, these adaptive behavioral responses may diverge from the conventional social standards [1]. We explore the interplay of behavioral and attachment disorders, examining how prenatal fentanyl usage interacts with neglect in shaping child development. The patient presented in this report exhibited manifestations indicative of disinhibited social engagement disorder, as delineated in DSM-V, which encompasses behaviors such as "a recurrent pattern wherein the child actively approaches and engages with unfamiliar adults" and "a discernible pattern characterized by extreme inadequacies in caregiving" [2]. Additionally, she was diagnosed with attention-deficit/hyperactivity disorder, oppositional defiant disorder, and disinhibited social engagement disorder.

The prevalence of nonmedical fentanyl use is high within populations characterized by a paucity of socio-economic resources, encompassing homelessness and food insufficiency [3]. Moreover, the abuse of fentanyl co-occurs with a spectrum of frequently abused substances, inclusive of morphine, methamphetamine/amphetamine, cannabinoids, and cocaine [4]. The utilization of the epidural administration of bupivacaine, lidocaine, and chloroprocaine, along with narcotics such as fentanyl, serves to minimize the necessary anesthesia dosage [5]. This approach aims to prevent sedation in newborns postdelivery due to potential placental transmission. Although the clinical application of fentanyl supplementation for pain management during the labor and delivery process remains devoid of adverse repercussions [4], extended gestational exposure to this agent imparts an augmented propensity for enduring detrimental behavioral consequences in progeny, manifesting as executive function impairments, attention-deficit/hyperactivity disorder (ADHD), and autism spectrum disorders [6]. Children at preschool and school-age junctures who have experienced prenatal exposure to opioids present below-average intelligence quotient (IQ) levels, languid language development, and attentional deficits [7].

Fentanyl exhibits rapid transplacental and fetal brain transfer [8]. Research reveals a notable surge in uterine blood flow post 12 weeks of gestation, potentially heightening transplacental drug passage, particularly for lipid-soluble substances, as their transfer rates are flow-dependent [8]. While this investigation does not specifically address fentanyl misuse, its findings are pertinent to unregulated drug utilization. The medical employment of fentanyl during pregnancy is presently classified as category C, meaning risk cannot be ruled out. It is critical to acknowledge the paucity of comprehensive and longitudinal studies examining the relationship between pregnant females, unregulated fentanyl use, and behavioral outcomes.

Prenatal substance misuse has long been recognized as a significant contributor to a range of physical and psychological impairments [9]. An investigation unveiled that children exposed to such substances are susceptible to neonatal abstinence syndrome (NAS) and exhibit low birth weight. Furthermore, children with prenatal drug exposure diagnosed with attention-deficit/hyperactivity disorder (ADHD) were commonly placed in foster care [10]. Reports have also documented the emergence of behavioral issues in children exposed to prenatal substance misuse, frequently manifesting during their school-age years [9]. In particular, the misuse of cocaine during pregnancy has been shown to disrupt the child's capacity for arousal and attention regulation [11]. Early intervention is paramount when dealing with severe ADHD, but a systematic review conducted in 2012 indicated that substance use and antisocial behavior were the least responsive to treatment; individuals with untreated symptoms experienced notably poorer outcomes [12].

Bowlby's monotropic theory posits that a child possesses an inherent need to establish a primary attachment with a single figure, termed monotropy, which plays a pivotal role in facilitating child development [13]. Bowlby's maternal deprivation hypothesis further underscores the critical nature of maintaining uninterrupted attachment between the infant and their primary caregiver, typically the mother, as continuous disruptions in this relationship may engender enduring cognitive, social, and emotional challenges in the child [13]. Such disruptions often manifest as attachment disorders, as defined in the DSM-V. Mary Ainsworth's attachment theory was developed after her time observing interactions in Uganda between infants and their primary caregivers. From her observations, she categorized three main attachment styles such as secure, anxious-ambivalent, and anxious-avoidant, shedding light on the impact of early relationships on lifelong emotional well-being and interpersonal dynamics [14]. The patient under consideration in this case experienced a protracted absence of a singular caregiver until 18 months of age, precipitating the onset of disinhibited social engagement disorder. Research has demonstrated that children placed into adoptive families prior to the age of six months exhibit developmental outcomes on par with their non-adopted siblings, whereas those adopted after this six-month threshold face an elevated risk of enduring cognitive deficits, disinhibited social behavior, inattention/overactivity, and autistic traits [15]. Notably, the patient in this case was placed for adoption well after the critical six-month mark and later developed disinhibited social engagement disorder and attention-deficit/hyperactivity disorder. Furthermore, the adverse effects of prenatal substance abuse add another layer of complexity to this clinical scenario.

## Conclusions

In conclusion, the case study presented underscores the likely impact of prenatal fentanyl exposure and disrupted attachment on child development and well-being. The interplay of behavioral and attachment disorders, exacerbated by prenatal substance misuse, highlights the urgent need for comprehensive intervention strategies. From a scientific perspective, the rapid transplacental transfer of fentanyl and its potential neurodevelopmental consequences underscore the gravity of addressing substance misuse during pregnancy. Furthermore, the patient's experience exemplifies the profound implications of early attachment disruptions on long-term developmental outcomes, emphasizing the importance of maintaining stable caregiver relationships during infancy. Moving forward, a concerted effort is warranted to address the systemic issues underlying substance misuse and inadequate caregiving, ensuring the provision of optimal support for vulnerable children and families. Through interdisciplinary collaboration and targeted interventions, we can strive toward mitigating the adverse effects of prenatal substance exposure and promoting healthier developmental trajectories for all children.
